# Cardiometabolic trajectories preceding dementia: a longitudinal nested case‐control study with 11 years of follow‐up

**DOI:** 10.1002/alz70860_104061

**Published:** 2025-12-23

**Authors:** Zimu Wu

**Affiliations:** ^1^ Monash University, Melbourne, VIC, Australia

## Abstract

**Background:**

Poor cardiometabolic health is a risk factor for cognitive decline and dementia. This study examined the trajectories of a range of cardiometabolic factors in the years leading up to dementia, compared to the trajectories among individuals without dementia.

**Method:**

A total of 1,078 dementia cases were matched to 4,312 controls, using data obtained from 19,114 community‐dwelling older individuals recruited in Australia and the United States. Body mass index (BMI), waist circumference (WC), systolic and diastolic blood pressure (SBP/DBP), glucose, high‐ and low‐density lipoprotein (HDL/LDL), triglycerides and total cholesterol were repeatedly measured over 11 years. Mixed‐effects models were used to model the retrospective trajectories of these factors.

**Result:**

Compared to controls, cases had lower BMI (*p* <0.002 for years ‐7 to 0) and WC (*p* <0.003 for years ‐10 to 0) close to a decade before dementia diagnosis, as well as a faster decline in BMI (*p* <0.001) and WC over time (*p* = 0.004). Compared to controls, cases generally had higher HDL levels (*p* = 0.03), in particular between 5 to 3 years before dementia (*p* = 0.001‐0.002) but with a decline in levels just before diagnosis. Dementia cases had consistently lower levels of SBP and triglycerides in the decade prior to diagnosis, as well as higher LDL and total cholesterol. However, the between‐group differences were minor, and not significant at any time point.

**Conclusion:**

Up to a decade before dementia diagnosis, there may be a decline in BMI, WC and HDL. These findings provide insights into cardiometabolic changes preceding dementia and the potential for early monitoring and intervention.

